# Electro-Oxidation–Plasma Treatment for Azo Dye Carmoisine (Acid Red 14) in an Aqueous Solution

**DOI:** 10.3390/ma13061463

**Published:** 2020-03-23

**Authors:** Héctor Barrera, Julián Cruz-Olivares, Bernardo A. Frontana-Uribe, Aarón Gómez-Díaz, Pedro G. Reyes-Romero, Carlos E. Barrera-Diaz

**Affiliations:** 1 Centro Conjunto de Investigación de Química Sustentable CCIQS, UAEM-UNAM, Carretera Toluca Atlacomulco, km 14.5, C.P. Toluca 50200, Estado de México, Mexico; hectorbarreragonzalez@gmail.com (H.B.); bafrontu@unam.mx (B.A.F.-U.); 2 Facultad de Química, Universidad Autónoma del Estado de México, Paseo Colón intersección Paseo Tollocan S/N, Toluca 50120, Estado de México, Mexico; jcruzolivares@gmail.com; 3 Instituto de Química, Universidad Nacional Autónoma de México, Circuito Exterior, Ciudad Universitaria, Ciudad de México 04510, Mexico; 4 Facultad de Ciencias, Universidad Autónoma del Estado de México, Campus El Cerrillo, Carretera Toluca - Ixtlahuaca Km 15.5, Piedras Blancas, Toluca 50200, Estado de México, Mexico; aaron_kfg@hotmail.com

**Keywords:** azo dye Carmoisine, advanced oxidation process, sequenced process, degradation, electro-oxidation, plasma at atmospheric pressure

## Abstract

Currently, azo dye Carmoisine is an additive that is widely used in the food processing industry sector. However, limited biodegradability in the environment has become a major concern regarding the removal of azo dye. In this study, the degradation of azo dye Carmoisine (acid red 14) in an aqueous solution was studied by using a sequenced process of electro-oxidation–plasma at atmospheric pressure (EO–PAP). Both the efficiency and effectiveness of the process were compared individually. To ascertain the behavior of azo dye Carmoisine over the degradation process, the variations in its physical characteristics were analyzed with a voltage–current relationship, optical emission spectra (OES) and temperature. On the other hand, chemical variables were analyzed by finding out pH, electrical conductivity, absorbance (UV/VIS Spectrophotometry), chemical oxygen demand (COD), cyclic voltammetry (CV), energy consumption and cost. The sequenced process (EO–PAP) increased degradation efficiency, reaching 100% for azo dye Carmoisine (acid red 14) in 60 min. It was observed that the introduction of small quantities of iron metal ions (Fe^2+^/Fe^3+^) as catalysts into the plasma process and the hydrogen peroxide formed in plasma electrical discharge led to the formation of larger amounts of hydroxyl radicals, thus promoting a better performance in the degradation of azo dye. This sequenced process increased the decolorization process.

## 1. Introduction

About 70% of the world dye production corresponds to azo compounds, which are characterized by one or various azo chromophores (–N=N–) attached to benzene and/or naphthalene rings with lateral –OH and –SO_3_H groups [1,2]. The most common azo dyes in the food industry are yellow—Tartrazine (E 102) and Sunset Yellow (E 110)—and red dyes—Azorubine (E122), Ponceau 4R (E124), Allura Red (E 129) and Amaranth (E123).

**Figure 1:**
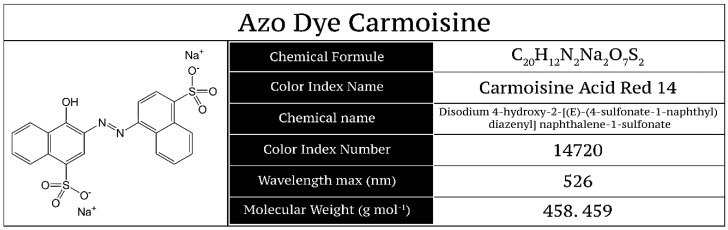
Chemical structure of azo dye Carmoisine (Acid Red 14 or additive E122).

The azo dye Carmoisine is classified as azorubine (E122) and is also called acid red 14 or additive E122 in the European Union (EU) [3]; the chemical structure of Carmoisine is shown in Figure 1. The use of this red dye in the food industry has increased because of the coloration of confectionery, jams, candies, jellies, ice cream, soft drinks, alcoholic beverages, etc.

Despite its extensive use, a number of negative effects on human health have been identified as a result of numerous studies [4,5]. It is known that the decay products of azo dyes (e.g., aromatic amines) are toxic and carcinogenic [6]. Moreover, recent publications have confirmed the interaction between azo dyes and human serum albumin [7] and hemoglobin [8]. According to some reports, synthetic food colorants may lead to super excitation and hyperactivity in children, especially when used in excess [9], as well as allergic and asthmatic diseases [10]. Furthermore, limited biodegradability in the environment has become a major concern about the removal of azo dyes.

International regulations established maximum permitted levels of azo dyes in wastewater from food and other industries; these dyeing effluents must be decolored before discharge. Many of these dyes are large, stable organic molecules, and, consequently, decolorization can involve either adsorption or degradation. 

There are various conventional techniques that are used in the industry such as trickling filters, active sludge, chemical coagulation, adsorption, ion exchange, and biological methods that have been studied extensively for the removal of azo dyes and many other organic compounds [11,12]. However, these methods are inefficient in removing azo dyes [13,14], since they have some disadvantages such as being nondestructive, producing large amounts of sludge, and, in some cases, treatment costs are high [12,15]. 

Over the last fifteen years, several electrochemical advanced oxidation processes (EAOPs) have been tested for the remediation of waters contaminated with organic pollutants [16,17,18,19]. They are based on the on-site generation of a strong oxidant-like hydroxyl radical (^•^OH), which can mineralize most organic compounds because of its high standard redox potential (E° = 2.80 V/SHE). The most ubiquitous EAOP is electrochemical oxidation (EO), in which organic pollutants are directly oxidized on the anode surface (M) and/or much more rapidly destroyed by an adsorbed hydroxyl radical M (^•^OH) produced via Reaction (1) at a high applied current [20,21,22,23]:

M + H_2_O → M(^•^OH) + H^+^ + e^−^ (1)

Likewise, plasma technology is also considered an AOP, and this promising technology is interesting because of its environmental compatibility and high removal efficiency. The electrical discharge generated in the plasma process leads to various physical and chemical effects such the primary formation of oxidizing species: radicals (^•^OH, H^•^, O^•^), molecules (H_2_O_2_, O_3_, etc.), cavitation sonic waves, ultraviolet light, and electrohydraulic cavitation [24].

The active species that are involved in the degradation of organic pollutants are mainly hydroxyl radicals and hydrogen peroxide. Hydroxyl radicals are aggressive and nonselective species that directly attack organic compounds, thus leading to the oxidation of these compounds. Additionally, in the presence of salt irons in the plasma process, hydrogen peroxide forms in-situ, and the electro-Fenton process produces large numbers of hydroxyl radicals [25]. 

In this context, coupled and sequenced processes are the most suitable technologies for the oxidative degradation of dyes and colorants, in which attention has been paid to mineralization: photo-catalysis [26,27], Fenton-Like oxidation [28], Fenton and photo-Fenton processes [29]. 

In the present study, a novel sequenced process of electro-oxidation with plasma at atmospheric pressure is suggested as a method to promote the decolorization and mineralization of azo dye Carmoisine (Acid Red 14) in an aqueous solution. To ascertain the behavior of physical variables, the following were analyzed: voltage and current, optical emission spectra (OES) and temperature. On the other hand, chemical variables were analyzed while considering pH, electrical conductivity, absorbance (UV/VIS spectrophotometry), chemical oxygen demand (COD), cyclic voltammetry (CV), energy consumption and cost. This process is an attractive and versatile alternative, since ^•^OH radicals are produced in high concentrations in water, thus maximizing the reaction rate and eliminating sludge production. 

## 2. Materials and Methods

### 2.1. Azo Dye Carmoisine (Acid Red 14) Solution

A test solution of azo dye Carmoisine was prepared with distilled water at a concentration of 5 mg·L^−1^ from a stock solution of azo dye Carmoisine (50 mg·L^−1^). The used azo dye Carmoisine was Sigma-Aldrich 98%. All the solutions were prepared with the use of amber glass volumetric material. A sample was taken to measure the wavelength (526 nm) at maximum absorbance. Real wastewater containing very low concentrations of Carmosine (below 1 ppm) is clearly visible because of the red coloration and causes serious deterioration in aqueous environments [30]; for this reason, this concentration was to be around this value chosen. Additionally, in order to use spectrophotometry as analytical technique, diluted solutions were required to be fitted to the Beer–Lambert law. 

### 2.2. Electro-Oxidation (EO) Treatment

**Figure 2:**
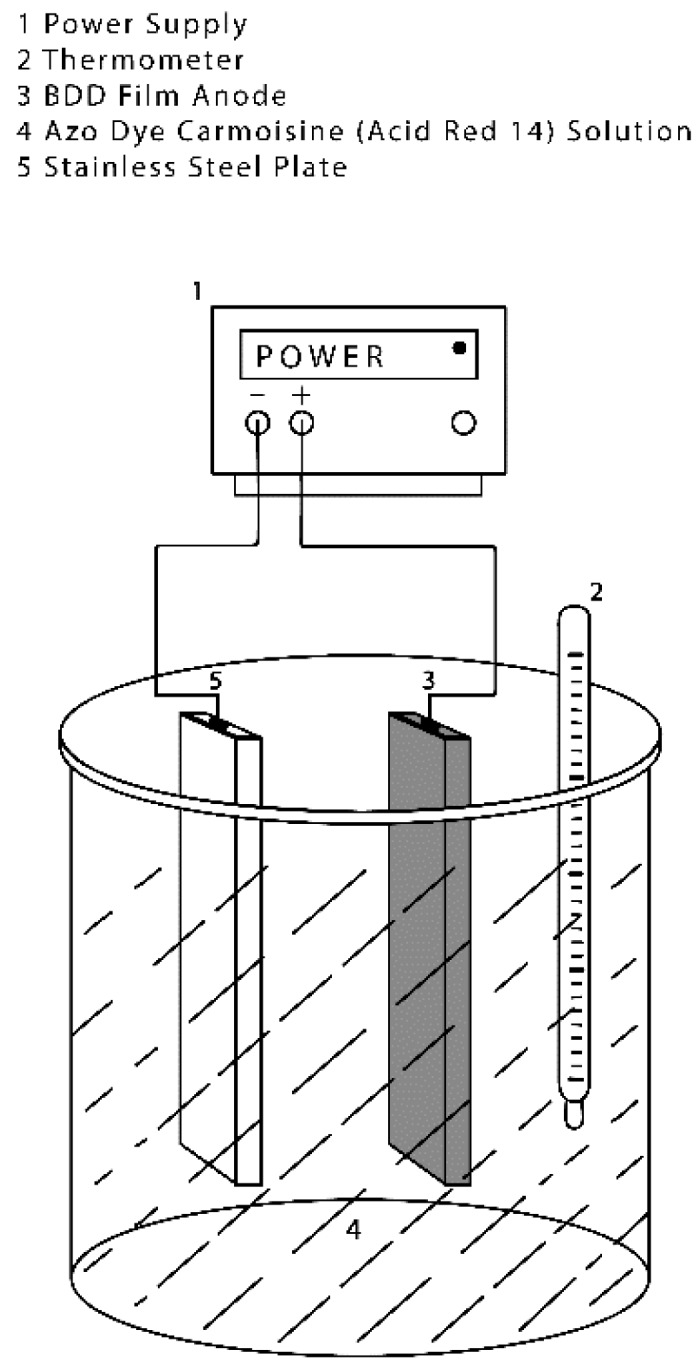
Schematic figure of electro-oxidation (EO) process for the degradation of the azo dye Carmoisine solution.

As shown in Figure 2, the electro-oxidation (EO) process was developed in a batch cylindrical electrochemical reactor (Glassware shop, Toluca, Mexico) that contained a stainless steel plate as a cathode and a BDD anode (boron-doped diamond film supported on a niobium substrate). The surface area of each electrode was 45 cm^2^ (2.5 cm × 18 cm). The 1.25 L reactor vessel was filled with 1.0 L of test solution containing 5 mg·L^−1^ of azo dye Carmoisine and 0.1 L of 0.01 M Na_2_SO_4_ as a supporting electrolyte; this solution was used in all the experiments. The reactor was fed by a direct current source with current values of 1.35, 0.90 and 0.45 amperes (30, 20 and 10 mA·cm^−2^, respectively).

All the experiments were carried out at room temperature (20 °C ± 5 °C), pH values of 5.2 ± 0.5, and in triplicate. The results show the average of three independent experiments. 

As part of the reactor design, a sampling faucet was adapted to the device, thus allowing for an easy opening and closing during the sampling in order to avoid any interference during the degradation process. Likewise, a magnetic stirrer bar was placed within the reactor to mix the solution of azo dye Carmoisine at a constant speed for all experiments.

At the initial stage of the degradation process of the azo dye Carmoisine by electro-oxidation (EO) treatment, an analysis by UV/VIS was conducted at different time intervals. Under the best conditions of pH, current density, and treatment time, the azo dye Carmoisine concentration was determined and analyzed with the use of chemical oxygen demand (COD) and Cyclic Voltammetry (CV).

### 2.3. Plasma Treatment at Atmospheric Pressure

**Figure 3:**
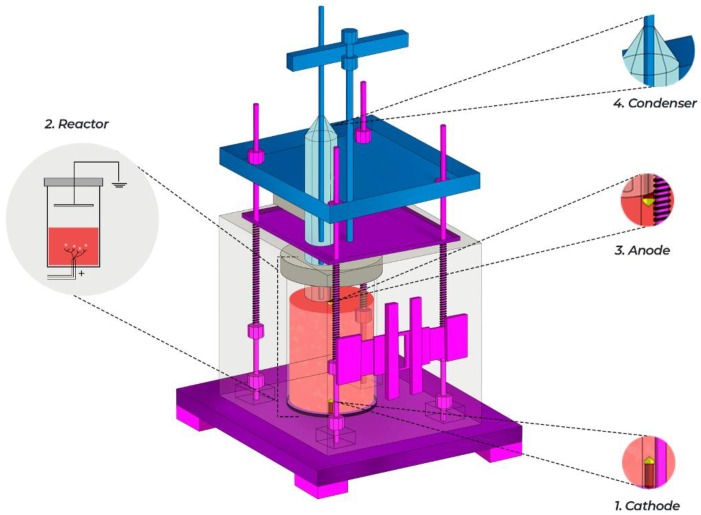
Experimental system of the plasma reactor at atmospheric pressure; frontal view.

The plasma treatment was performed in a reactor (Glassware shop, Toluca, Mexico) (Figure 3) made of borosilicate glass with capacity of 1 L and equipped with two tungsten electrodes of 3 mm diameter each that were aligned perpendicularly; the cathode was immersed in the solution, and the anode was on the solution surface. The distance from the anode to the surface solution was set at 5 mm, and the cathode had a length of 1 cm inside. This reactor was adapted with an optical fiber (Ocean Optics, wavelength range in 200–1100 nm) to collect the light generated from the discharge and monitored by optical emission spectroscopy. To relieve the pressure generated within, a condenser was placed at the top of the system reactor, as well as a temperature sensor and voltage connections. The treatment involved the generation of plasma by an electric discharge on the solution surface at atmospheric pressure, using a DC power supply model Keysight N8937A (Budd Lake, NJ, USA), with range of 4 kV and 50 mA; the voltage applied between the electrodes was 1.2 kV, and the current was 40 mA and was over the entire treatment maintained. To ensure a steady current in the process, the breakdown was monitored by an oscilloscope (Tektronix, TDS 3014B, Beaverton, OR, USA).

The sample for this treatment was prepared in a 1 L test solution of azo dye Carmoisine at a concentration of 5 mg·L^−1^ and with 0.045 mM FeSO_4_/H_2_SO_4_ as a catalyst and supporting electrolyte. Treated samples were taken at regular intervals of 10 min for 120 min, and they were equally analyzed to determine the physical and chemicals variables of the individual treatments. All the experiments were carried out at pH values of 5.2 ± 0.5 and in triplicate.

### 2.4. Electro-Oxidation–Plasma Sequenced Treatment

For the sequenced electro-oxidation–plasma process, a 1.0 L test solution was prepared. It contained 5 mg·L^−1^ of azo dye Carmoisine and 0.1 L of 0.01 M Na_2_SO_4_ as a supporting electrolyte, using the electrolysis system described above. In the electro-oxidation process a direct-current power source supplied the system with 1.35 A, which corresponded to a current density of 30 mA·cm^−2^. The experiments were conducted at room temperature (20 °C ± 5 °C) and initial pH = 5.2 ± 0.5 for 30 min. 

After this treatment, the resulting solution underwent a plasma treatment for 30 min by using the system designed to carry out this process. Samples were taken at regular intervals to determine physical and chemical variables. 

### 2.5. Analysis by Chemical Oxygen Demand (COD)

For COD analysis, samples were processed with the use of 0.7–40 mg·L^−1^ HACH^®^ digestion vials in a Thermo Scientific^®^ Orion COD125 reactor digestion (Beverly, MA, USA), and they were subsequently measured in a HACH DR 5000 according to American Public Health Association (APHA) standard procedures and method (Equation (2)) [31]; all the samples were analyzed in triplicate.

% COD removal = [(CODC_i_ – CODC_f_/ CODC_i_)] × 100 (2)

### 2.6. Energy Consumption and Cost

The energy consumption per volume of effluent treatment was estimated and expressed in kW·h ·dm^−3^ [32]. In the case of electro-oxidation, the plasma and sequenced treatment were taken into consideration for the estimation of energy consumption following Equation (3).

EC = [∆E_c_It]/1000V (3)

where t is the time of electrolysis (h), ∆E_c_ is the average cell voltage (V), I is the electrolysis current (A), and V is the sample volume (dm^−3^). 

Considering the cost of electrical energy around $0.15 USD per kW·h, the value required to degrade a unit volume of effluent is given by the following Equation (4).

Cost (US$·dm^−3^) = EC (kW·h·dm^−3^) × 0.15 (US$/kW·h) (4)

### 2.7. UV/VIS Spectrophotometry

The analysis by UV/VIS was conducted with a HACH*^®^* DR6000 UV/VIS Spectrophotometer (Loveland, CO, USA) by applying a wavelength of 350–700 nm for the direct measure of absorbance.

The samples were scanned in a quartz cell with a 1 cm optical path by triplicate. The calibration curve obtained a coefficient of correlation (r^2^) of 0.999. The decolorization of the azo dye Carmoisine solutions was monitored from the absorbance (A) decay at its maximum absorption wavelength, 526 nm, determined from the spectra. The color removal efficiency or percentage of color removal was then calculated as follows (Equation (5)):

Color removal (%) = (A_0_−At/A_0_) × 100 (5)

where A_0_ and At denote the absorbance at initial time and after an electrolysis time t, respectively.

### 2.8. Cyclic Voltammetry (CV)

CV analysis was performed with an azo dye Carmoisine solution at a concentration of 5 mg·L^−1^, by the use of a standard three-electrode cell. Waveform and potential were applied with a potentiostat–galvanostat Autolab 302N (Utrecht, The Netherlands) software NOVA 2.1. Measurements were conducted before and after treatments by using a BDD working electrode, a platinum counter electrode, and Ag/AgCl (3 M KCl) as reference in a 10 mL one-compartment electrochemical cell. The voltammetric cell was kept at 20.0 ± 0.5 °C. Oxygen was removed by bubbling pure nitrogen through the solution for 10 min.

Two different procedures for the electrochemical pre-treatment of the surface were carried out in supporting electrolytes, pH 5.2 0.01 M sodium sulfate, before every electrochemical assay:

- Anodic pre-treatment: A positive potential was applied for 30 min, Eap = +3.0 V. The BDD surface was oxidized, Ox-BDD, together with long and extensive oxygen evolution.
- Cathodic pre-treatment: A negative potential was applied for 30 min, Ecp = −3.0 V. The BDD surface was reduced, Red-BDD, together with long and extensive hydrogen evolution.

### 2.9. Hydrogen Peroxide Test Kit HACH^®^

Determinations of hydrogen peroxide residues during the reaction were performed with the use of a HACH® High Range Test kit (Loveland, CO, USA). This method is based on the fact that H_2_O_2_ oxidizes iodide to iodine in the presence of acid and a molybdate catalyst. The iodine formed is titrated with a thiosulfate solution, incorporating a starch indicator. To validate the used method, a certificate of all the analysis performed from the raw material was received from the HACH^®^ Company. Component 2291700 lot 7012, 7016 & 7015, date of expiration June 2021.

## 3. Results and Discussion

### 3.1. Electro-Oxidation (EO) Treatment

For the electro-oxidation treatment, a series of preliminary tests were conducted based on pH and current density to ascertain the operating conditions of this process. COD was assessed to measure the effect of these variables on the degradation of azo dye Carmoisine (Acid Red 14) for different time intervals. According to the literature, a solution’s pH is a factor that influences the degradation rates of pollutants in many processes, as it affects the capacity of adsorption and the dissociation of the target compound, catalyst surface change, the oxidation potential of valence band, and other physiochemical properties [33]. 

Two different pH values, 5.2 and 9.0, were selected. The best results obtained for COD removal efficiency were 87.3% and 77.03% for the values of 5.2 and 9.0, respectively, after 120 min of treatment by using 30 mA·cm^−2^. This process can be favored by the generation of highly oxidant species such as the hydroxyl radical ^●^OH on the anode at pH = 5.2; this pH is favored due to the fact that azo dye shows a brilliant red color at pH values close to neutrality, and acidic or basic solutions change its color due to the protonation of the sulfonic groups or the deprotonation of the phenol moiety.

**Figure 4:**
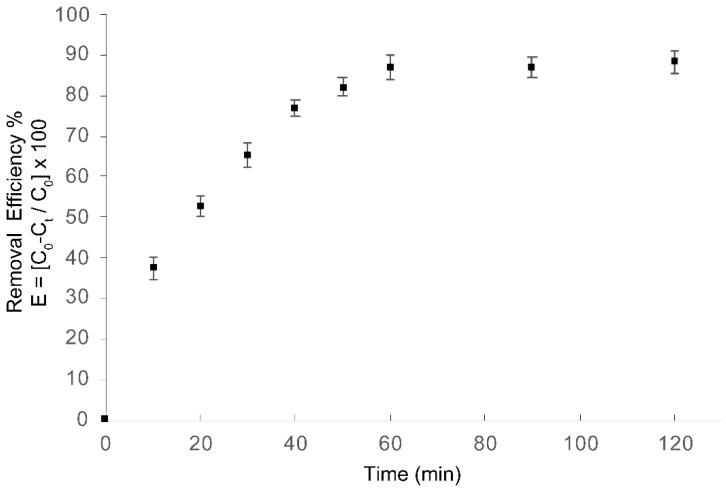
Chemical oxygen demand (COD) removal efficiency for azo dye Carmoisine (5 ppm) as a function of treatment time. (▪) Electro-oxidation (EO) process. The current density was 30 mA·cm^−2^, the pH of the sample was adjusted to 5.2. Treated volume 1.1 L.

In this manner, to evaluate the effect of current density in azo dye Carmoisine (Acid Red 14) degradation, three different current densities of 10, 20 and 30 mA·cm^−2^ were applied at pH = 5.2 and monitored by COD analysis. The results showed a degradation of 87.3% for 30 mA·cm^−2^, 63.03% for 20 mA·cm^−2^, and 41.03% for 10 mA·cm^−2^. Therefore, COD removal efficiency improved with the increase of current density. Thus, the electrochemical process was assessed based on the best experimental conditions of pH = 5.2 ± 0.5 and a current density of 30 mA·cm^−2^ over 60 min, achieving a COD removal of 86.9% under these conditions, as shown in Figure 4. When the electrolysis time was longer than 60 min, the process of electro-oxidation was not favored, since COD removal slightly increased to 87.3%.

The instantaneous current density was determined by COD removal (ICD-COD) [34], obtaining 16.69% at 30 mA·cm^−2^, 18.07% at 20 mA·cm^−2^, and 23.74% at 10 mA·cm^−2^. According to the literature, these low values indicate there was a greater energy supply than that required to accomplish the degradation of the molecule. This is explained by the fact that the degradation of Carmoisine occurred on the surface of the anode (direct oxidation), and, therefore, it lost current efficiency. Additionally, the low concentration (5 ppm) of the dye used in the aqueous solution affected the current efficiency [35]. Hence, an increase in the concentration of azo dye Carmoisine may lead to a greater current efficiency. 

### 3.2. Plasma at Atmospheric Pressure (PAP) Treatment

Plasma treatment as a part of AOP’s has been successfully applied for the remediation of pollutants in wastewater. Using the operating conditions found for the electrochemical treatment, such as pH and volume, UV/Vis, COD and CV analyses were performed in order to monitor the degradation of azo dye Carmoisine (Acid Red 14) at different time intervals over plasma treatment. 

A non-thermal plasma treatment is an important source of oxidant reactive species such as ^●^OH, H^•^, and O^•^, as well as long-life active molecules such as H_2_O_2_ and O_3_, excited-state neutral molecules, and ionic species, which can oxidize organic pollutants. The addition of Fe^+2^ or Fe^+3^ to the aqueous medium significantly accelerates the degradation rate of organics because of the additional formation of ^•^OH, which generates H_2_O_2_ from Fenton’s reaction [36]. The azo dye Carmoisine achieved an 86.70% rate of removal in 60 min. However, the rate of the plasma process was significantly improved when the treatment time reached 90 min, achieving a COD removal of 94.2%. When the plasma treatment time was longer than 90 min, COD removal slightly increased to 95.5%. 

**Figure 5:**
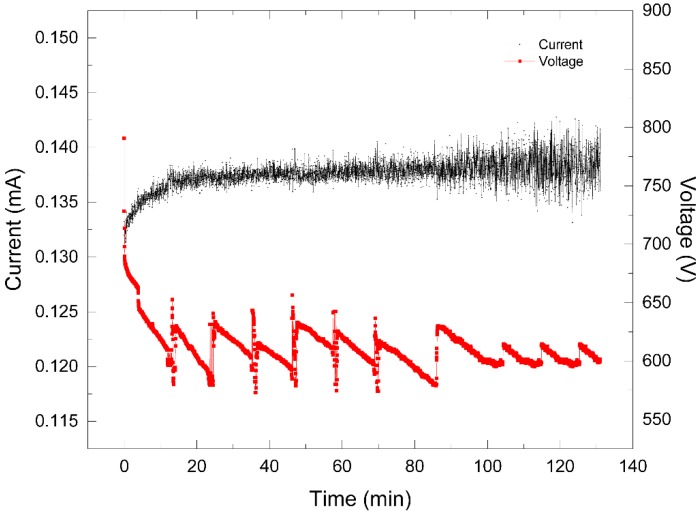
Graphic behavior of voltage and current during plasma at atmospheric pressure (PAP) treatment for azo dye Carmoisine (5 ppm) as a function of electrical measurement. The pH of the sample was adjusted to 5.2. The treated volume was 1.0 L. The current is black line, and the voltage is the red line.

On the one hand, a direct current source fed the plasma reactor with the use of a DC power supply applying a voltage of 1.2 kV and with a current limited to 130 mA. When the discharge was generated, a decrease in the voltage level was observed until 680 V was reached, which corresponded to an increase in the current of 130 mA. Thus, the interaction between the plasma and the sample, after the reaction was initiated, led to a decrease in the voltage while the current increased. This behavior could be attributed to the ion production in the liquid sample, thus allowing for a greater current flow. Figure 5 shows the response of voltage after such a reaction was initiated, in contrast with the increase in the current. In the first 12 min, the voltage decreased and the current increased. The peaks that subsequently appeared were the result of settings in the separation distance from the water surface to the high voltage tip and a decrease in the level of the liquid sample due to evaporation by heating. This setting in the gap between the tip and the interface kept the electrical power constant.

### 3.3. Electro-Oxidation–Plasma at Atmospheric Pressure (EO–PAP) Sequenced Treatment

**Figure 6:**
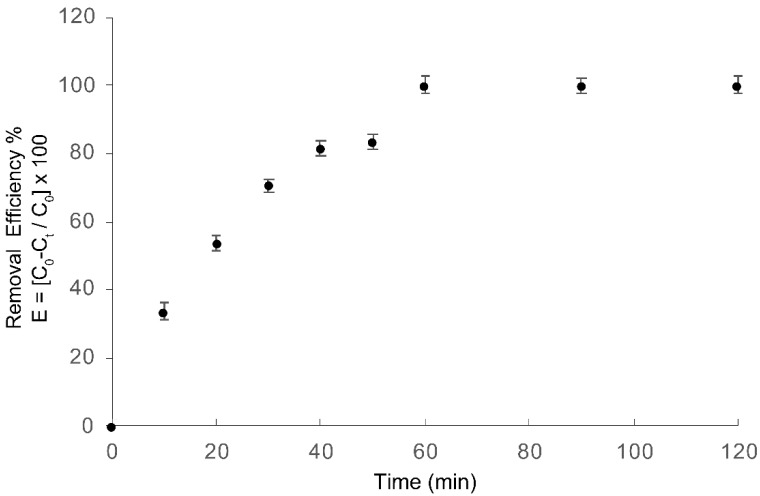
COD removal efficiency for azo dye Carmoisine (5 ppm) as a function of treatment time. (●) EO–PAP sequenced treatment (30 min of EO + 30 min of PAP). The pH of the sample was adjusted to 5.2. The treated volume was 1.0 L.

Figure 6 shows COD reduction as a function of treatment time for azo dye Carmoisine, obtaining a 100% COD removal after 60 min of treatment. The EO–PAP sequenced process showed better results in the degradation rates than those obtained with single treatments.

**Figure 7:**
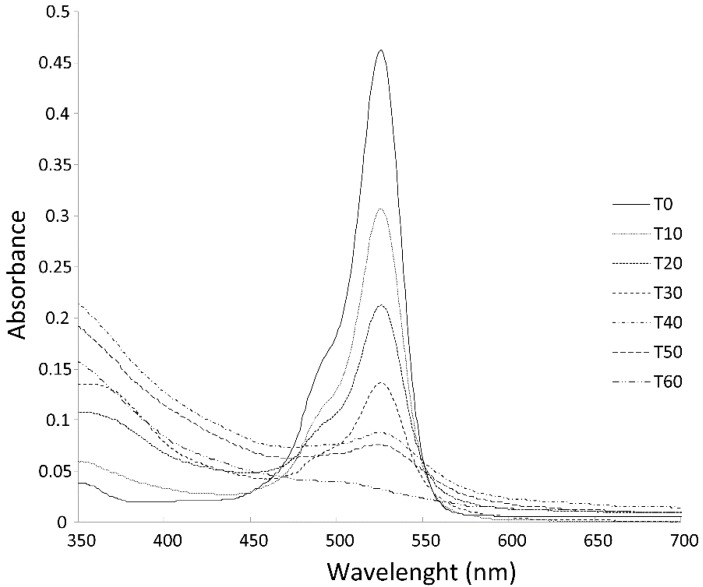
Effect of the type of treatment analyzed by UV/Vis. EO–PAP sequenced treatment (30 min of EO + 30 min of PAP) after 60 min of treatment of azo dye Carmoisine (5 ppm). The pH of the sample was adjusted to 5.2. The treated volume was 1.0 L.

These results suggest that the sequenced primarily treatment led to a decolorization of azo dye Carmoisine, which was observed in the UV/Vis spectra by the decrease in the wavelength at the highest absorbance peak (526 nm). This behavior is attributed to the electrochemical formation of ^●^OH and S_2_O_8_^2−^ species which initiate the first step in the mechanism of this oxidation process and involves benzene, naphthalene, indane, indole and benzofuran derivatives, thus generating rapidly short chain carboxylic acids [37]. These were no longer detectable in the UV/Vis spectra (Figure 7). On the other hand, the solution in the plasma treatment and the combination treatment solution contained Fe (II) ions, which were used with the H_2_O_2_ formed in the plasma process to generate the Fenton reagent. Fe salts are unstable above pH = 6 due to the formation of hydroxide species that precipitate.

**Figure 8:**
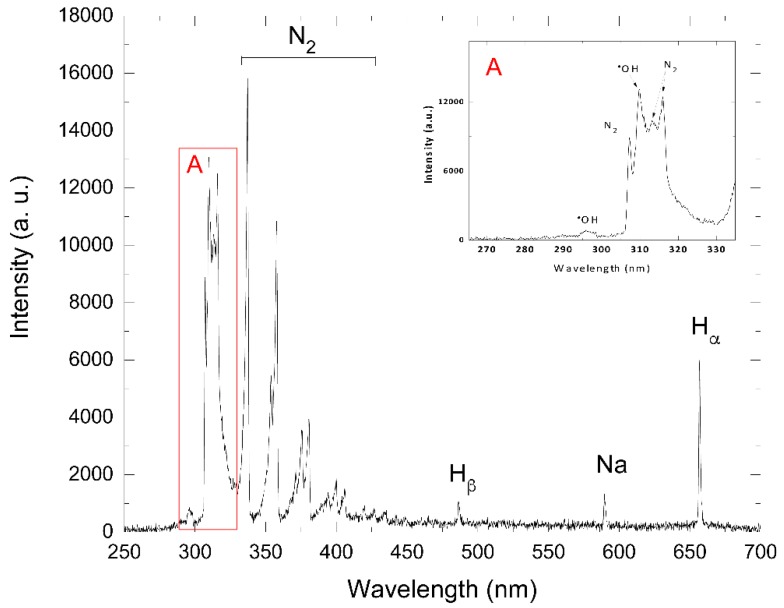
Optical spectrum of plasma emission in the air–liquid interface during the EO–PAP sequenced treatment for azo dye Carmoisine (5 ppm). The pH of the sample was adjusted to 5.2. The treated volume was 1.0 L. The insert shows the absorption spectra of the detection of ^●^OH radicals.

Furthermore, the mineralization process of azo dye Carmoisine occurred in the second part of the sequenced treatment when the dye solution was treated by AOP oxidation, thus generating highly oxidizing species such as ^●^OH radicals, which are observed in Figure 8 via the spectra of optical emission in the air–liquid interface. These spectra also show the formation of other elements such as N_2_, H_ß_, H_α_, and Na, as shown in Table 1. 

The determination of hydrogen peroxide in the final solution corroborated the mineralization of azo dye Carmoisine, obtaining 3 mg·L^−1^ H_2_O_2_ residual. Additionally, because it is a strong oxidant (E° = 1.77 V), it can be produced via the recombination of the ^●^OH radical, especially in the case of plasma technologies. Hydrogen peroxide does not significantly react with most organic compounds, at least at appreciable rates for water treatment. Nevertheless, H_2_O_2_ increases the collective oxidizability of the plasma and significantly affects plasma chemistry. In presence of H_2_O_2_, many more ^●^OH radicals can be directly or indirectly via various reactions (e.g., dissociation, photolysis, and metal metal-based catalytic reactions) generated [24].

This analysis also showed that the reaction followed a first-order kinetic model (R^2^ = 0.8214) and a value for the constant rate of k = 0.0235 min^−1^.

### 3.4. Active Species Reactivity over EO–PAP Sequenced Treatment

In order to support the degradation of azo dye Carmoisine, it is necessary to understand the model for oxidation of organics on BDD; organics are effectively destroyed (electrochemical mineralization) by the large amounts of hydroxyl radicals (^•^OH) produced on the BDD surface by water oxidation, as seen in Equations (6)–(8) [38].

BDD + H_2_O → BDD (^●^OH) + H^+^+ e^−^ (6)

R + BDD (^●^OH) → BDD + RO + H^+^+ e^−^ (7)

BDD organics (^●^OH) → BDD + ½ O_2_ + H^+^+ e^−^ (8)

The supporting electrolytes also contribute to the degradation of the dye; in this case, Na_2_SO_4_ was oxidized by ^●^OH to form S_2_O_8_^2−^ (as seen in Equation (9)), which is a strong oxidant (E° = 2.01 V) and actively contributes to the degradation of azo dye Carmoisine [39].

^●^OH + SO_4_^2−^ → S_2_O_8_^2−^ + ^●^OH (9)

Furthermore, in the plasma process, there are several active species involved in the reaction mechanism, beginning with the Equation (10), where plasma in solution generate species such as ^●^OH and highly energized electrons. On the other hand, the hydroperoxyl radical and hydroxyl radical can promote the generation of hydrogen peroxide (E° = 1.77 V) which, in the reaction, promotes organic degradation (see Equation (11)). In addition, the residual H_2_O_2_ concentration of the plasma process with ferrous ion and ferric ion (Equation (12)) are lower than those in the plasma process without iron ions [40].

Plasma + H_2_O → e^−^ + H* + ^●^OH (10)

HO_2_^●^ + ^●^OH → H_2_O_2_ (residual) + O_2_ (11)

Fe^3+^ → Fe^2+^ (plasma/Fe^3+^ process) (12)

This indicates that iron ions (including ferrous ions) can sufficiently utilize the residual hydrogen peroxide and produce a larger amount of the reactive non-selective radical (^●^OH) (Equation (13)). The generation of the radicals entails a complex reaction sequence in an aqueous solution (Equation (14)) [41].

Fe^2+^ + H_2_O_2_ (residual) → Fe^3+^ + OH^−^ + ^●^OH (13)

^●^OH + Fe^2+^ → OH^−^ + Fe^3+^ (14)

Moreover, the newly formed ferric ions may catalyze hydrogen peroxide, causing it to be decomposed into water and oxygen. Ferrous ions and radicals are also formed in the reactions. The reactions are as shown in Equations (15)–(18), and these reactions of hydrogen peroxide with ferric ions are referred to as Fenton-like [42,43,44].

Fe^3+^ + H_2_O_2_ → Fe(OOH)^2+^ + H^+^ (15)

Fe(OOH)^2+^ → Fe^2+^ + HO_2_^●^ (16)

Fe^2+^ + HO_2_^●^ → Fe^3+^ + HO_2_^−^ (17)

Fe^3+^ + HO_2_^●^ → Fe^2+^ + O_2_ + H^+^ (18)

As noticed in Equation (19), H_2_O_2_ can act as an ^●^HO scavenger as well as an initiator (Equation (13)).

^●^HO + H_2_O_2_ → H_2_O + HO_2_^●^ (19)

A hydroxyl radical can oxidize organics (R) through the abstraction of protons, thus producing organics radicals (^●^R), which are highly reactive and can be further oxidized (Equation (20) [45].

RH + ^●^OH → H_2_O + ^●^R (20)

As a result, the parallel degradation routes of organic compounds are triggered by the interaction of these highly oxidant species, which are present during sequenced treatment (EO–PAP).

### 3.5. Optical Emission Spectroscopy (OES)

The luminescence of the corona discharge that interacts with the samples with azo dye Carmoisine was by optical emission spectroscopy in the atmosphere–air interphase observed and analyzed. In this type of electrical discharge, lines and bands are observed due to the interaction of plasma and water, which generates reactive species such as ^●^OH, N_2_, H_β_, Na, and H_α_. Figure 8 shows the generation of these species, which were produced through electronic impact processes such as N_2_ (313.6, 315.93, 337.13, 353.67, 357.69, 371.05, 375.54, 380.49, 394.30, 399.926, and 405.94 nm) belonging to the second positive system (C_3_Π_u_ → B_3_Π_g_). In the case of positive groups or bands between 316 and 380 nm, their presence is related to luminescence in the positive column of the electric discharge. This second positive system band can usually be found in nitrogen discharges or atmospheric discharge plasmas; the latter can be obtained in a laboratory. Using a simplified collisional radiative model, the reactions and the species formed in the process of interaction are in Table 1 listed. H_β_ and H_α_ (486.13 and 656.79 nm), whose ionization process goes from n = 2 to n = 4 and n = 2 to n = 3, respectively; these hydrogens are related to the excitation energy of the radiated decay. The observed species depended on the composition of the atmosphere where the plasma was generated; in this case, the experimental system contained water and air vapor (nitrogen and oxygen). Unlike the emergence of highly reactive species such as the ^●^OH radical observed in the emission lines at 295.12, 307.8, and 308.9 nm, which are related to the oxidation properties of the solution, this was formed by the interaction of plasma with the dye solution. Species such as ^●^OH, $A^{2}\Sigma^{+}-A^{2}\Pi$, are shown in Table 1. This could have been related to the presence of H_2_ in the air discharge, giving rise to an increase in the dissociation processes of the nitrogen, hydrogen, and oxygen molecules with other species forming (NO, ^●^OH, and NH). The ^●^OH species were formed according to the following Equations (21) and (22):

H_2_ + e (4.3 eV) → H + H + e^−^ (21)

H + O_2_ → ^●^OH + O^●^ (22)

### 3.6. Cyclic Voltammetry

**Figure 9:**
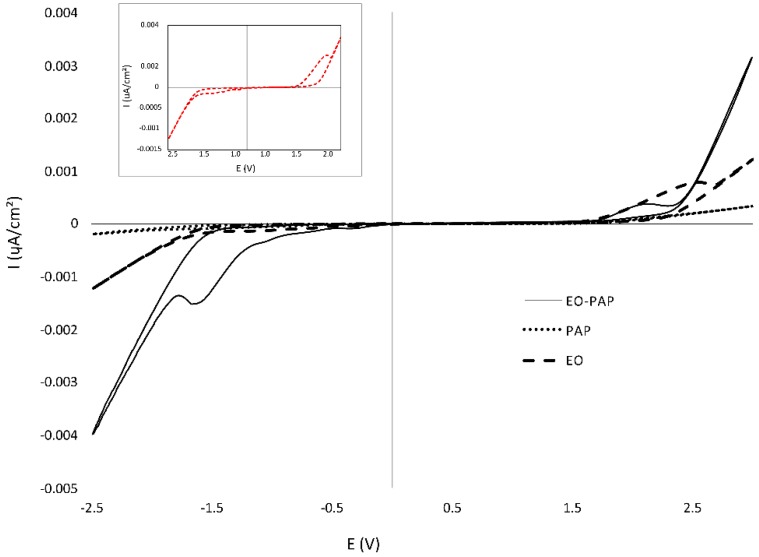
Cyclic voltammetry of 5 mg·L^−1^ of azo dye Carmoisine in 0.05 M H_2_SO_4_ after three process (EO/PAP and EO–PAP). The scan rate was 100 mV·s^−1^. Electrodes CE: Pt, RE: Ag/AgCl, WE: BDD.

The analysis of electrochemical behavior for each process (Figure 9) was assessed through the efficiency of the electrochemical, plasma and sequenced process (EO–PAP) by cyclic voltammetry during the mineralization of azo dye Carmoisine.

A series of assays were conducted with cyclic voltammetry by using a three-electrode system with platinum (counter electrode), Ag/AgCl (reference electrode), and BDD (working electrode). After the electrochemical process, the results indicated an anodic peak corresponding to the oxidation of azo dye Carmoisine, detectable at 1.5–2.0 V (Figure 9; insert). This peak represented the direct electrochemical oxidation of the dye present in the system. In contrast, when cyclic voltammetry was applied after a plasma process, this peak significantly decreased, which shows that the solution of Carmoisine was almost fully degraded. However, the results after the sequenced process (EO–PAP) revealed that an anodic peak corresponding to the oxidation of azo dye Carmoisine was still detectable at 2.05 V as a small peak. This observed behavior was a consequence of the conditions of the process. Therefore, the first stage in the degradation process was the direct oxidation on the BDD electrode and the generated intermediates, which had higher oxidation potentials, were oxidized at later stages of the electrolysis on the BDD electrode by the ^●^OH radicals produced at the surface. Behavior for the anodic oxidations of azo dye Carmoisine has been reported and has been found to be very similar to the compound studied here.

These cyclic voltammetry analyses in conjunction with the results of UV-Vis and COD verified the efficiency of the degradation of azo dye Carmoisine compound at the end of the sequenced process (EO–PAP).

### 3.7. Estimation of Energy Consumption

The adoption of AOP technology should consider some important aspects to provide a feasible process, such as the selection of the anode, the quantity of electrolyte support, energy consumption and operational cost. These last two elements are of great importance in implementation at a large scale. 

**Figure 10:**
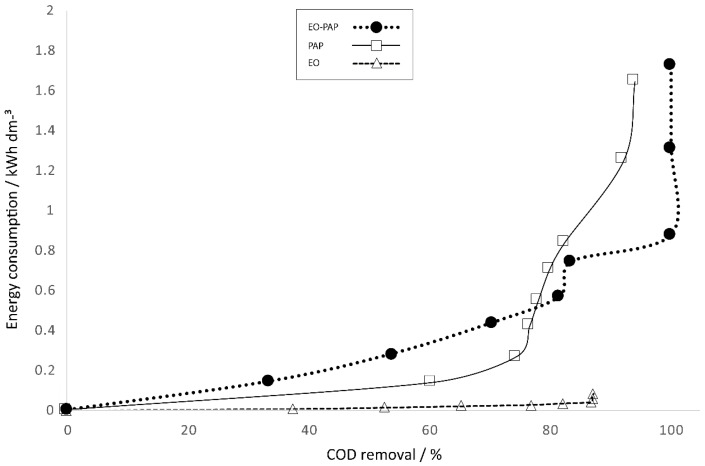
COD removal efficiency for azo dye Carmoisine (5 ppm) as a function of energy consumption. (△) EO, (□) PAP and (●) EO–PAP sequenced processes. The current density was 30 mA·cm^−2^, and the pH of the sample was adjusted to 5.2. The treated volume was 1.1 L.

A different behavior was observed in all of the treatments when we utilized the EO treatment with BDD as an anode while applying a voltage of 25.8 V (30 mA·cm^−2^) (Figure 10). We noticed the lowest energy consumption with a higher COD removal in comparison with the results accomplished with the plasma process (610 V and 135 mA, respectively). These data concur with those reported in the literature, in which it has been demonstrated that electrochemical treatment decreases energy consumption and cost.

On the other hand, the study of the EO–PAP sequenced process showed an increased (17.60%) COD degradation, with the azo dye Carmoisine reaching higher degradation in comparison with the electro-process and plasma process in a time of 60 min with an energy consumption of 0.8771 kW·h·dm^−3^.

## 4. Conclusions

The sequenced EO–PAP process under optimized conditions has proven to be an effective and promising technology in the abatement of azo dye Carmoisine in an aqueous solution. Because of the improvement achieved in the mineralization of the dye, obtaining 100% removal in 60 min, this treatment can be an attractive alternative to degrade more complex molecules by virtue of the active species’ reactivity generated in situ. Future work in our laboratory points to the use of real water from agricultural systems that contain herbicides, fertilizers and pesticides. Thus, this point can represent an important advantage. The addition of iron ions as a catalyst during the plasma process induced Fenton-like reactions and enhanced the degradation of azo dye Carmoisine (acid red 14) when it was fused with a sequenced study. This is attributed to the formation of hydrogen peroxide, since it reacts with ferric or ferrous iron, thus producing a hydroxyl radical that actively contributes to the degradation of molecule azo dye Carmoisine (acid red 14). The attack to the chromophore group of the molecule of azo dye Carmoisine causes a decolorization of the dye that is increased by the end of the sequenced process, showing a complete fading.

## Figures and Tables

**Table 1:** Electronic transitions species oxidative generates during the PAP process.

Species	Predicted Wavelength (nm)	Experimental Wavelength (nm)	System/Transition	v′-v″
^●^OH	295.12	295.538	3064 Å System $A^{2}\Sigma^{+}-A^{2}\Pi$	3–2
^●^OH	307.8	307.172	3064 Å System $A^{2}\Sigma^{+}-A^{2}\Pi$	0–0
^●^OH	308.9	309.935	3064 Å System $A^{2}\Sigma^{+}-A^{2}\Pi$	0–0
N_2_	313.6	313.122	Second positive system $C^{3}\Pi_{u}-B^{3}\Pi_{g}$	2–1
N_2_	315.93	315.881	Second positive system $C^{3}\Pi_{u}-B^{3}\Pi_{g}$	1–0
N_2_	337.13	337.246	Second positive system $C^{3}\Pi_{u}-B^{3}\Pi_{g}$	0–0
N_2_	353.67	353.656	Second positive system $C^{3}\Pi_{u}-B^{3}\Pi_{g}$	1–2
N_2_	357.69	357.641	Second positive system $C^{3}\Pi_{u}-B^{3}\Pi_{g}$	0–1
N_2_	371.05	371.24	Second positive system $C^{3}\Pi_{u}-B^{3}\Pi_{g}$	2–4
N_2_	375.54	375.413	Second positive system $C^{3}\Pi_{u}-B^{3}\Pi_{g}$	1–3
N_2_	380.49	380.622	Second positive system $C^{3}\Pi_{u}-B^{3}\Pi_{g}$	0–2
N_2_	394.3	394.126	Second positive system $C^{3}\Pi_{u}-B^{3}\Pi_{g}$	2–5
N_2_	399.926	399.926	Second positive system $C^{3}\Pi_{u}-B^{3}\Pi_{g}$	1–4
N_2_	405.94	405.922	Second positive system $C^{3}\Pi_{u}-B^{3}\Pi_{g}$	1–3
H_β_	486.13	486.44	n=4−n=2	
Na		589.64	3s–3p	
H_α_	656.79	656.98	n=3− n=2	
